# 缓解期存在的克隆性造血对伴NPM1突变急性髓系白血病患者化疗后造血恢复的影响

**DOI:** 10.3760/cma.j.issn.0253-2727.2023.10.009

**Published:** 2023-10

**Authors:** 淋淋 王, 苏宁 陈

**Affiliations:** 1 苏州大学附属第一医院，江苏省血液研究所，国家血液系统疾病临床医学研究中心，国家卫生健康委员会血栓与止血重点实验室，苏州 215006 National Clinical Research Center for Hematologic Diseases, Key Laboratory of Thrombosis and Hemostasis of Ministry of Health, Jiangsu Institute of Hematology, the First Affiliated Hospital of Soochow University, Suzhou 215006, China; 2 徐州医科大学盐城临床学院盐城市第一人民医院，盐城 224000 The Yancheng Clinical College of Xuzhou Medical University, the First People's Hospital of Yancheng, Yancheng 224000, China

**Keywords:** 白血病，髓系，急性, 克隆性造血, NPM1突变, 造血恢复, Leukemia, myeloid, acute, Clonal hematopoiesis, NPM1 mutation, Hematopoietic recovery

## Abstract

**目的:**

探讨缓解期存在的克隆性造血（CH）对伴NPM1突变急性髓系白血病（AML）患者化疗后造血恢复的影响。

**方法:**

回顾性分析2016年7月至2019年6月苏州大学附属第一医院收治的初诊伴NPM1突变AML患者86例，对患者诊断时的临床资料、二代测序检测结果和缓解期骨髓基因突变检测结果进行分析。应用Log-rank方法比较造血恢复的差异，采用单因素及多因素Cox比例风险模型分析影响造血恢复的因素。

**结果:**

86例AML伴NPM1突变患者中位年龄50（15～69）岁，男39例，女47例，41例患者给予“7+3”强化疗方案诱导治疗，45例患者给予含低剂量阿糖胞苷的低强度方案诱导治疗。86例患者诊断时最常见的突变为FLT3、DNMT3A、TET2、IDH1、IDH2，缓解期存在CH相关突变患者21例，为DNMT3A、TET2、ASXL1、IDH1/IDH2基因突变。缓解期存在CH相关突变组患者的中性粒细胞恢复时间与缓解期无CH组患者比较差异无统计学意义（*P*＝0.282），但前者的血小板恢复时间明显延长［26（95％*CI* 21～32）d对25（95％*CI* 23～26）d，*P*＝0.032］。单因素Cox比例风险模型分析提示年龄、诱导化疗方案、缓解期存在CH相关突变为影响血小板恢复的危险因素，多因素Cox比例风险模型分析提示诱导化疗方案（*HR*＝0.454，*P*＝0.001）、缓解期存在CH相关突变（*HR*＝0.520，*P*＝0.027）为影响血小板恢复的独立危险因素。

**结论:**

缓解期存在的CH使AML伴NPM1突变患者化疗后血小板恢复延迟。

## Abstract (en)

**Objective:**

To investigate the effect of clonal hematopoiesis（CH）in remission on hematopoiesis recovery in patients with NPM1 mutated acute myeloid leukemia（AML）after chemotherapy.

**Methods:**

Retrospective analysis was performed on 86 patients with NPM1^mut^ AML newly diagnosed and treated in the First Affiliated Hospital of Soochow University between July 2016 and June 2019. Their clinical data and NGS test results at diagnosis were analyzed. Moreover, bone marrow samples in remission were tested using Sanger sequencing. The log-rank test was used to analyze the difference in hematopoietic recovery, and Cox proportional hazard models were used to analyze the prognostic factors affecting hematopoietic recovery.

**Results:**

The median age of the 86 NPM1^mut^ AML patients was 50 years（15–69 years）. There were 39 males and 47 females. Forty-one patients were induced with intensity chemotherapy（“7 + 3”）, whereas 45 patients were treated with low-dose cytarabine-based induction chemotherapy. At diagnosis, The most common mutations in the patients were FLT3, DNMT3A, TET2, and IDH1/IDH2 mutations. CH-associated mutations persisted in 21 patients during remission, and the mutations were DNMT3A, TET2, ASXL1, and IDH1/IDH2. The recovery time of neutrophils in patients with CH-associated mutations in remission was consistent with that in patients without CH in remission（*P*＝0.282）but the recovery time of platelets in patients with CH in remission was significantly longer［26（95％ *CI* 21–32）days *vs* 25（95％ *CI* 23–26）days, *P*＝0.032］. Furthermore, univariate analysis indicated that age, induced chemotherapy program, and CH in remission were risk factors for platelet recovery, whereas multivariate analysis indicated that induced chemotherapy program and CH in remission were independent risk factors for platelet recovery（*HR*＝0.454, *P*＝0.001 and *HR*＝0.520, *P*＝0.027, respectively）.

**Conclusion:**

CH in remission delays the hematopoietic recovery of patients with NPM1^mut^ AML after chemotherapy.

急性髓系白血病（AML）患者NPM1突变发生率约为30％，NPM1突变AML患者预后相对较好，诱导化疗缓解率高，但部分患者完全缓解（CR）期仍可检测到白血病前克隆，常见的克隆性造血（Clonal hematopoiesis, CH）分子突变为DNMT3A、TET2和ASXL1（DTA）基因突变，SRSF2、IDH1/IDH2基因突变亦可在缓解期持续存在，并对白血病复发及患者生存产生影响[1]–[6]。达到形态学缓解的患者通常在化疗开始后第4周血细胞逐渐恢复，但有部分患者血细胞恢复明显延迟。缓解期存在的CH对AML伴NPM1突变患者化疗后造血恢复的影响尚不清楚。本研究对86例AML伴NPM1突变患者进行回顾性分析，旨在探讨影响该亚组患者化疗后造血恢复的因素。

## 病例与方法

1. 病例资料：回顾性分析2016年7月至2019年6月于苏州大学附属第一医院初诊的所有AML伴NPM1突变患者148例，排除早期死亡患者7例，2个疗程仍未达形态学缓解患者12例，第1次CR（CR_1_）期无骨髓标本患者43例，本研究纳入86例患者，中位年龄50（15～69）岁，男39例，女47例。根据缓解期是否存在CH相关基因突变，将86例患者分成两组：缓解期无CH相关突变65例，缓解期存在≥1个CH相关突变21例。41例患者给予“7+3”联合或不联合地西他滨/索拉菲尼诱导化疗，45例患者给予含低剂量阿糖胞苷的低强度方案联合或不联合地西他滨/索拉菲尼诱导化疗，缓解期存在CH相关突变组患者与不存在CH相关突变组患者诱导化疗方案差异无统计学意义（*P*＝0.312）。

2. 基因突变检测：对诊断时的骨髓样本中分离的DNA进行髓系血液疾病51或176种靶向基因组二代测序（Illumina），对缓解期骨髓样本中分离的DNA采用Sanger测序检测诊断时存在的突变，其中FLT3-ITD/TKD采用毛细管电泳法。

3. 造血恢复定义：造血恢复的界值定义为CR伴部分血液学恢复（CR with partial hematologic recovery，CRh）[7]–[9]，即中性粒细胞恢复时间定义为从化疗开始到化疗结束后中性粒细胞绝对计数达到0.5×10^9^/L，血小板恢复时间定义为从化疗开始到化疗结束后PLT达到50×10^9^/L。

4. 统计学处理：应用卡方检验和方差分析比较缓解期存在CH相关基因突变组患者与不存在组患者的临床特征及基线资料。应用Log-rank方法比较造血恢复的差异。双侧检验*P*<0.05为差异有统计学意义。单因素及多因素Cox比例风险模型分析影响造血恢复的因素。纳入的因素包括年龄、ELN2017危险分层、粒细胞缺乏（粒缺）期是否存在感染、骨髓抑制期IL-11/TPO或G-CSF/GM-CSF的应用、化疗方案、缓解期是否存在CH相关突变。以上统计学处理均应用SAS9.4软件。

## 结果

1. 患者的临床特征：本研究纳入86例AML伴NPM1突变患者，缓解期无CH相关突变65例，缓解期存在≥1个CH相关突变21例，中位年龄分别为48岁和52岁。缓解期存在CH相关突变组CR率较低（66.7％对93.8％，*P*＝0.004），且治疗前DNMT3A、DNMT3A-R882、TET2基因突变发生率更高，差异均有统计学意义（*P*<0.05）。两组临床资料比较详见表1。

**表1:** 86例伴NPM1突变急性髓系白血病（AML）患者临床特征及基线资料

特征	缓解期无CH相关突变组（65例）	缓解期存在≥1个CH相关突变组（21例）	*χ*^2^值/*F*值	*P*值
性别（例，男/女）	30/35	9/12	0.067	0.792
年龄[（岁，*M*（范围）]	48(15~69)	52(21~69)	2.980	0.088
AML类型[例（%）]				
原发	63(96.9)	21(100.0)	0.662	1.000
继发	2(3.1)	0(0)		
WBC[×10^9^/L，*M*（范围)]	15.1(0.9~331.2)	50.4(1.3~253.0)	1.550	0.217
HGB[g/L，*M*（范围)]	88(46~149)	87(50~119)	0.070	0.798
PLT[×10^9^/L，*M*（范围)]	73(8~713)	66(35~313)	0.000	0.979
骨髓原始细胞[%，*M*（范围)]	64(20~94)	58(23~91)	0.090	0.770
ELN 2017危险分层[例（%）]				
良好	47(72.3)	14(66.7)	0.298	0.719
中等	16(24.6)	6(28.6)		
差	2(3.0)	1(4.8)		
诱导化疗方案[例（%）]				
低强度化疗	32(49.2)	13(61.9)	1.022	0.312
强化疗	33(50.8)	8(38.1)		
诱导化疗疗效[例（%）]				
CR	61(93.8)	14(66.7)	8.216	0.004
CRi	4(6.2)	7(33.3)		
治疗前基因突变[例（%）]				
FLT3	36(55.4)	16(76.2)	2.874	0.090
FLT3-ITD	25(38.5)	8(38.1)	0.001	0.976
DNMT3A	19(29.2)	14(66.7)	9.406	0.002
DNMT3A-R882	9(13.8)	11(52.4)	11.135	0.001
TET2	13(20.0)	9(42.9)	4.356	0.037
IDH1	13(20.0)	2(9.5)	0.592	0.442
IDH2	12(18.5)	2(9.5)	0.390	0.532
PTPN11	10(15.4)	1(4.8)	0.795	0.373
KRAS/NRAS	8(12.3)	4(19.0)	0.170	0.680
WT1	4(6.2)	0(0)	1.355	0.568
GATA2	4(6.2)	1(4.8)	0.056	1.000
CEBPA	1(1.5)	2(9.5)	3.006	0.146
ASXL1	0(0)	1(4.8)	3.132	0.244
粒细胞缺乏期感染[例（%）]	59(90.8)	19(905)	0.000	1.000
骨髓抑制期应用G-CSF/GM-CSF[例（%）]	36(55.4)	14(66.7)	0.830	0.362
骨髓抑制期应用IL-11/TPO[例（%）]	31(47.7)	14(66.7)	2.291	0.130

注 CH：克隆性造血；ELN：欧洲白血病网；CR：完全缓解；CRi：完全缓解伴不完全血液学恢复；TPO：血小板生成素

2. 诊断时和缓解期的基因图谱：86例NPM1突变AML患者诊断时最常见的共突变基因为FLT3、DNMT3A、TET2、IDH1、IDH2等，缓解期存在CH相关突变患者21例，缓解期存在的CH相关突变基因为DNMT3A、TET2、ASXL1、IDH1/IDH2（图1）。

**图1:**
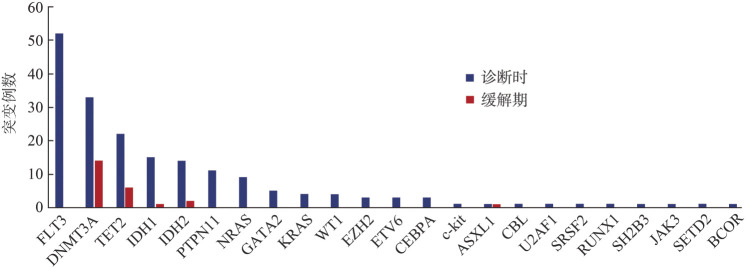
86例伴NPM1突变急性髓系白血病患者诊断时及缓解期的基因突变谱

3. 缓解期存在的CH相关突变对造血恢复的影响：缓解期存在CH相关突变组患者的中性粒细胞恢复时间与缓解期无CH相关突变组患者差异无统计学意义（*P*＝0.282），但缓解期存在CH相关突变组患者的血小板恢复时间明显延长［26（95％*CI* 21～32）d对25（95％*CI* 23～26）d，*P*＝0.032］（图2A）。DTA亚组与无CH相关突变组比较亦有相同结果［27（95％*CI* 21～36）d对25（95％*CI* 23～26）d，*P*＝0.011］（图2B）。

**图2:**
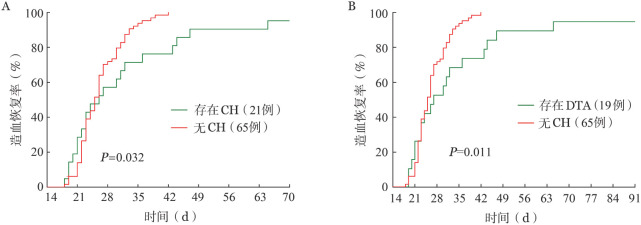
缓解期克隆性造血（CH）对伴NPM1突变急性髓系白血病患者化疗后血小板恢复的影响 A CH的影响； B DTA的影响 注 DTA：DNMT3A、TET2和ASXL1基因突变

4.诱导化疗后影响血小板及粒细胞恢复的因素：影响化疗后血小板恢复的单因素及多因素分析见表2，单因素分析结果显示，年龄、诱导化疗方案（低强度化疗）、缓解期存在的CH相关突变为影响化疗后血小板恢复的不良因素。上述因素纳入多因素Cox比例风险模型，结果显示诱导化疗方案（低强度化疗）、缓解期存在CH相关突变为影响化疗后血小板恢复的独立危险因素。影响化疗后粒细胞恢复的单因素及多因素分析见表3，单因素分析结果显示，年龄、诱导化疗方案（低强度化疗）可能为影响化疗后粒细胞恢复的不良因素，而G-CSF/GM-CSF的应用可能加速粒细胞恢复。上述因素纳入多因素Cox比例风险模型，结果显示诱导化疗方案（低强度化疗）为影响化疗后血小板恢复的独立危险因素。

**表2:** 影响伴NPM1突变急性髓系白血病患者化疗后血小板恢复的单因素及多因素分析

变量	单因素分析			多因素分析		
	*HR*	95%*CI*	*P*值	*HR*	95%*CI*	*P*值
年龄	0.983	0.968～0.997	0.022			
ELN危险分层	1.564	0.956～2.559	0.075			
粒细胞缺乏期感染	1.387	0.631～3.052	0.416			
IL-11/TPO	1.284	0.832～1.982	0.258			
化疗方案	0.480	0.305～0.755	0.002	0.454	0.285～0.724	0.001
缓解期CH突变	0.559	0.316～0.987	0.045	0.520	0.291～0.928	0.027

注 ELN：欧洲白血病网；TPO：血小板生成素；CH：克隆性造血

**表3:** 影响伴NPM1突变急性髓系白血病患者化疗后粒细胞恢复的单因素及多因素分析

变量	单因素分析			多因素分析		
	*HR*	95%*CI*	*P*值	*HR*	95%*CI*	*P*值
年龄	0.986	0.971～1.001	0.076			
ELN危险分层	1.147	0.700～1.878	0.586			
粒细胞缺乏期感染	1.392	0.634～3.055	0.410			
G-CSF/GM-CSF	1.543	0.992～2.398	0.054			
缓解期CH突变	0.768	0.452～1.304	0.328			
化疗方案	0.478	0.306～0.748	0.001	0.478	0.306～0.748	0.001

注 ELN：欧洲白血病网；CH：克隆性造血

## 讨论

AML患者化疗后造血恢复的影响因素较多，有文献报道长期化疗损伤造血祖细胞、基质祖细胞，化疗后给予自体骨髓基质细胞或间充质干细胞可加速造血功能的恢复[10]–[12]。一些刺激造血的药物可加快骨髓造血恢复，如rhG-CSF可促进化疗后粒细胞的恢复[13]，rhlL-11和rhTPO可加快血小板的恢复[14]–[15]。而粒缺期重症感染可能损伤造血干细胞（HSC）功能，抑制骨髓造血，使造血恢复延迟[16]。本研究中单因素分析提示rhG-CSF/GM-CSF的应用可能加速粒细胞的恢复。

本研究中52例（60.5％）患者伴有FLT3基因突变，FLT3基因是早期造血生长因子受体基因，与其配体结合后而被激活，FLT3发生二聚化，进而激活下游信号通路，如PI3K-AKT、JAK-STAT和RAS-MAPK等，对造血干/祖细胞的增殖和分化起调节作用，FLT3基因对AML的发生发展起重要作用[17]，但单因素及多因素分析显示FLT3基因突变并不影响急性白血病患者化疗后血小板和粒细胞的恢复（*P*值分别为0.170和0.716）。

本研究中，缓解期存在的CH相关突变为DNMT3A、TET2、ASXL1、IDH1/IDH2，此前文献中亦有报道[1]–[6],[18]。CH使造血恢复延迟的机制可能有以下方面：tet甲基胞嘧啶双加氧酶2（tet methylcytosine dioxygenase 2, TET2）是HSC自我更新和分化的关键调节因子，TET2缺乏导致5hmC的基因组水平下降，从而影响了HSC的自我更新、增殖和分化[19]；DNMT3A基因突变阻断了HSC分化[20]–[22]，DNMT3A突变体可以通过多克隆抑制复合物1（polycomb repressive complex 1, PRC1）阻断HSC的分化[23]；ASXL1基因突变使HSC的表观基因组发生改变，改变了HSC的功能，并增加白血病转化的易感性[24]。

CH的存在影响造血功能的恢复，单因素及多因素分析结果均显示缓解期存在CH相关突变为化疗后血小板恢复的独立影响因素，而CH相关突变对中性粒细胞的恢复有无影响仍存在争议。本研究中多因素分析CH相关突变对粒细胞对恢复无影响，Murphy等[25]报道，缓解期存在CH相关突变可使化疗后粒细胞及血小板恢复延迟，可能与纳入标准不同有关，他们的研究排除了需要接受二次诱导化疗或接受G-CSF治疗的患者。本研究的单因素分析提示rhG-CSF/GM-CSF的应用可能加速粒细胞的恢复，而rhG-CSF的应用是否可克服CH相关突变对化疗后粒细胞恢复的影响仍有待进一步探讨。

单因素分析提示高龄为影响血小板及粒细胞恢复的不良因素，尽管老年与CH的关联原因仍不完全清楚，但AML患者CH相关突变的发生率远远超过了相应年龄组的健康人群，尤其是>60岁的老年患者[26]–[27]。并且老年往往选择低强度化疗方案，而多因素分析显示低强度方案使AML伴NPM1突变患者的粒细胞及血小板恢复延迟。但随着越来越多靶向药物的上市，含靶向药物的化疗方案（如维奈克拉联合去甲基化药物等）是否可克服上述因素对造血恢复的影响，仍有待进一步探索。
